# Controversial membrane structure, independently verified after 22 years, is diagnostic and prognostic for cancer risk

**DOI:** 10.1007/s12551-026-01449-4

**Published:** 2026-06-22

**Authors:** Carolyn Mountford, Tyrone Humphries

**Affiliations:** 1 Institute for Biomedicine and Glycomics, Griffith University, Gold Coast Campus, Southport, QLD 4215 Australia; 2 DatChem Pty Ltd, Brisbane, QLD 4000 Australia

**Keywords:** Membranes, Two-dimensional correlated spectroscopy (2D COSY), Neutral lipid domains, Relaxation rates, Breast cancer risk, Fucosylated glycans

## Abstract

The history behind the proof, and acceptance, of neutral lipid domains in the plasma membranes of transformed and cancer cells is noteworthy. Not only for the controversy, but because this membrane structure has become a hallmark for women at risk for breast cancer. The membrane model, proposed in 1988 to contain isotropically tumbling domains, made up of triglycerides, cholesterol, and cholesterol ester, was not verified until 2010 by a team of Danish scientists. In the intervening 22 years, focus in this area was on the fluid lipid bilayer and the presence of intracellular droplets. The proof of neutral lipid domains in the plasma membranes, mobile on the MR timescale, involved chemists, physicists, pathologists, and surgeons from Australia and Canada and finally Denmark. Today, the appearance of these neutral lipid domains is the first of a series of changes that are diagnostic for risk of breast cancer and considered by some as a point of clinical pharmaceutical intervention for those at risk of developing cancer.

**Supplementary Information:**

The online version contains supplementary material available at https://doi.org/10.1007/s12551-026-01449-4.

## Preface

Five people contributed to the thought processes of the original observation. In order: **Rodney Porter (Oxford)** “When tissue becomes diseased, or there is an immune response, there is increased chemical activity.” **R. J. P. Williams (Oxford)** “Take the advantage of thinking like a chemist.” **Ian C. P. Smith (Canada)** “Molecules must be active on the NMR timescale to be seen in cells.” **Myer Bloom (Canada)** “The physical properties of membranes control their biological roles.” **Peter Russell (Sydney)** “Biological states exist that are not morphologically manifest and disease is often a multi-step process and histopathology cannot always discern all of the steps involved.”

## Early history

This work began in 1980 when immunologist Professor Tony Basten from Sydney used an animal model to investigate leukemic transformation. Using proton nuclear magnetic resonance (^1^H NMR) spectroscopy, changes at a molecular level were recorded when genetically programmed leukemic cells proliferated in the thymus of AKR mice (Mountford et al. 1980). These spectral changes were attributable to changes in membrane composition with increased molecular motion, which in turn reflect the stage of differentiation and the type of transformation of the lymphocyte lines, i.e., leukemic transformation. The data strongly suggested that ^1^H NMR spectroscopy was reporting on chemical changes as the preleukemic period ends and the development of the neoplasia starts. Since histopathology requires at least 1000 lymphoma cells for detection, it was unlikely that this number of cells was present. Thus, there had to be chemical activity taking place that was not discernible by light microscopy. This was the working hypothesis of pathologist Professor Peter Russell (Russell et al. 1998).

Today, these membrane changes are recognized as the first in a series of cellular changes, using a two-dimensional MR spectroscopy protocol on a clinical MR scanner (Ramadan et al. 2015; Santamaría et al. 2022). This observation has led to the concept that cells could be identified in transition towards a cancer developing.

## Singer and Nicolson membrane was not consistent with ^1^H NMR of stimulated or cancer cells

The phospholipid bilayer, proposed by Singer and Nicolson in 1972 (Nicolson 2013), was widely accepted as the standard for understanding membrane structure. In the 1980 s, there was a great number of solid-state NMR studies underway to investigate the structure and dynamics of the lipid bilayers, which in turn was integral for understanding how membrane proteins functioned (Auger 2017).

The results from studying cancer cells and models of tumor development suggested that the Singer and Nicolson model needed modifications. The studies reported below demonstrated that the presence of neutral lipid domains in cancer cell plasma membranes, as determined by ^1^H NMR spectroscopy, had similar chemical and physical characteristics to the very low-density lipoproteins (Williams et al. 1985), in that there were neutral lipids isotropically tumbling in the plasma membrane. A combined Australian and Canadian team investigated this hypothesis for a number of years.

## Proton NMR spectroscopy of cells

**Fig. 1:**
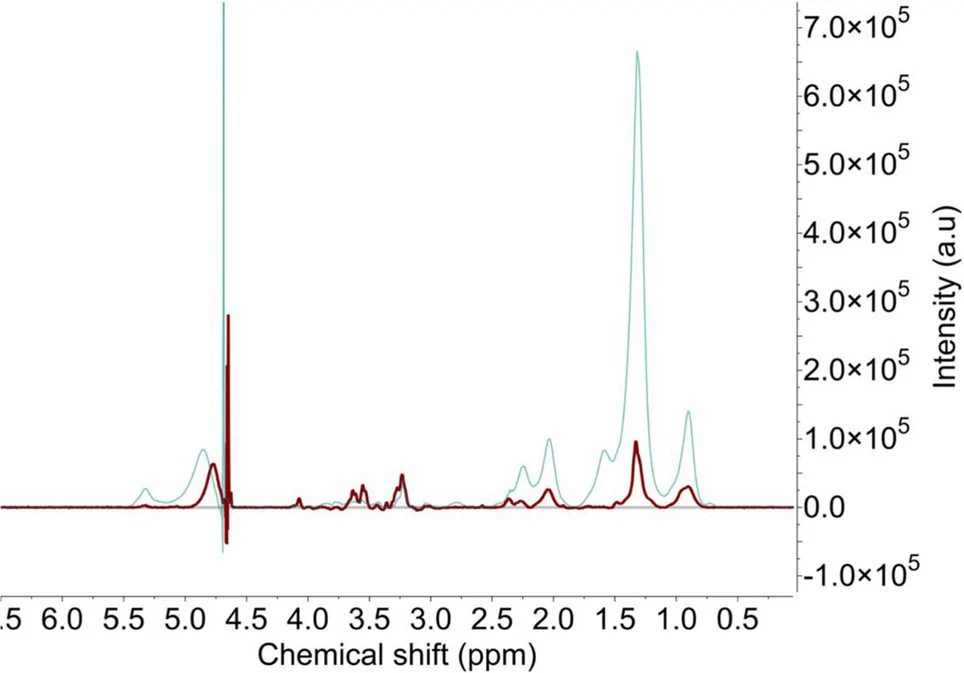
Representative 1D ^1^H NMR spectroscopy spectra of kidney cortex (bottom) and clear cell renal cell carcinoma (top) tissue. Tissue donor was a 56-year-old male undergoing radical nephrectomy for a kidney lesion. The lesion was diagnosed post nephrectomy as grade 3 ccRCC by hospital pathologists. Kidney cortex was taken from the same kidney at a region distal to the lesion. NMR spectroscopy was performed using an Avance Neo 400 NMR spectrometer (Bruker) on Topspin version 4.0.5 software. Spectra were acquired using a standard Bruker 1D pulse sequence with continuous wave presaturation of the water resonance during the relaxation delay for water suppression. Signal intensities used for quantitative analyses were measured from the spectra and subsequently normalized to the respective tissue weights. The spectra are shown as representative examples acquired and processed using identical parameters. Characteristic lipid resonances were observed at 0.89 ppm (methyl groups), 1.33 ppm (methylene groups), and 5.22 ppm (olefinic groups) (unpublished data)

Malignant cells and human biopsies all generated the same result, narrow-lined lipid spectra not recorded in most healthy tissues. Figure 1 provides a representative example of modern spectra from biopsied malignant and healthy kidney tissue. The spectra shown in Fig. 1 are representative examples were acquired using identical parameters. Quantitative signal intensities were measured from the spectra and subsequently normalised to tissue weight to account for differences in sample mass. The prominent resonances at 0.89, 1.33 and 5.22 ppm are consistent with mobile neutral lipid species that have previously been assigned to plasma membrane-associated lipid domains containing triglycerides, cholesterol and cholesterol esters. This assignment is supported by studies demonstrating membrane localisation of signals , the absence of correlation with intracellular lipid , and the chemical identification of neutral lipids in highly purified plasma membrane preparations (Wright et al. 1986; Mackinnon et al. 1989; Mackinnon et al. 1992). Finding the origin of these signals was the initial challenge. Cells available for study were cultured, grown, and maintained in a laboratory setting. The use of cell lines, that were well documented biologically, allowed for the study of how genetic changes occurring during tumor development affected the NMR spectrum.

One of the more important findings was that exposing stimulated lymphocytes and cancer cells to gadolinium nitrate saw the narrow lipid resonances broaden (Mountford et al. 1980). Since gadolinium did not pass through the membrane, the signals needed to be either in the membrane or on the surface of the cell. It turned out to be both (Mountford et al. 1982, 1987; Wright et al. 1986). The cancer cells had mobile lipid domains in the plasma membrane. They could also shed some of the mobile lipid signal.

Importantly, ^1^H NMR spectroscopy was able to distinguish between tumor cells with metastatic potential and those that produced only locally invasive tumors (Mountford et al. 1984a, b). This represented the first report of a prognostic molecular marker in a primary tumor. Specifically, a molecule that gave rise to a resonance with a long T2 relaxation rate (over 700ms), at the chemical shift of around 1.27 ppm, and clearly identified those primary tumors with the capacity to metastasize.

This created significant complexity in interpreting and understanding the role of the lipids as there were resonances with a relatively short T2 of less than 150 ms and others of over 700 ms (Mountford et al. 1984a, b) in the same spectral region.

The significance of the molecule with the longer T2 relaxation value was demonstrated when the metastatic capability of cells was reduced by treatment with the enzyme fucosidase (Wright et al. 1988). Following one generation of cell doubling in culture, the long T2 relaxation signal disappeared (Mountford et al. 1984a, b). Thus, the molecule with the long T2 relaxation value appeared to be a fucosylated molecule.

## Explaining the highly mobile lipid in the plasma membrane was the next challenge

The collaboration with Canadians Myer Bloom and Ian C. P. Smith provided the expertise to develop a working hypothesis to test.

It had been established that the narrow ^1^H NMR resonances observed in the methylene region of spectra from cancer cells and biopsies could be resolved into multiple components using Lorentzian-Gaussian deconvolution, allowing the measurement of transverse relaxation for individual signals (Delikatny et al. 1993). In subsequent measurements, non-exponential relaxation which is indicative of several components could be distinguished and shown to differ in cells with established biological behavior. Furthermore, by applying selective and non-selective measurements of longitudinal relaxation, a distinction between the various possible sources of the narrow resonances was made (Mountford et al. 1984a, b; Delikatny et al. 1993).

There were two potential mechanisms proposed by Myer Bloom, to account for narrow lines associated with protons in the system (Bloom et al. 1986; Holmes et al. 1987). The first involved lipid molecules located at positions in the plasma membrane such that the local normal to the bilayer surface makes an angle θ with the external magnetic field close to the ‘magic angle’, *θ*_m_ = cos^−1^(1/√3), at which the rapid reorientation motions and conformational transitions can average the intra-molecular dipolar interactions to zero. Since the local normal is the symmetry axis for the rapid reorientational motions, the intra-molecular dipolar linewidth had to be proportional to (3cos^2^θ−1) in membranes (Wennerstrom and Ulmius 1976; Bloom et al. 1977; Lepore et al. 1992).

The second possibility was that the narrow lines were quasi-Lorentzian in nature (Wennerstrom and Ulmius 1976; Bloom et al. 1978), arising from small structures which retain their integrity within, or in close association with, the plasma membrane for a sufficiently long time to tumble isotropically and produce narrow resonances distinct from those of the membrane lipids. This was the model most consistent with knowledge at the time.

## Two-dimensional proton NMR spectroscopy of cells

Implementing two-dimensional correlated spectroscopy (2D COSY) to provide unambiguous assignment of these lipids was the next important step. The first attempt at recording 2D COSY of a cell suspension took place in a school of chemistry, where it was initially regarded as an inefficient use of magnet time and raised biosafety concerns due to the placement of clinical specimens in the magnet. The 2D COSY of cells yielded a high-resolution magnetic resonance (MR) spectrum of lipid, cholesterol, and metabolites (Cross et al. 1984). Two-dimensional MR methods were successfully employed to simplify the problems of resonance assignment. The mobile lipids were confirmed to be triglycerides with cholesterol present (May et al. 1986). The 2D COSY data provided a plethora of important information.

## Time domains and window functions in the processing of two-dimensional COSY

The effect of altering time domains and window functions in 2D COSY spectra of biological specimens (Delikatny, Hull et al. 1991) was integral to the cell, biopsy, and human data processing of the next decades. This was undertaken by the William (Bill) Hull and Jim Delkatney, another Canadian, from the Bloom Lab. This development proved to be a most important step for understanding cell models and membrane structure and facilitated filtering out molecules according to their relaxation rates. Today, it is integral for the post processing of 2D COSY data collected in a clinical scanner.

The choice of window functions during post processing was shown to have dramatic effects on the resolution, sensitivity, and appearance of 2D spectra of cell specimens. For magnitude-mode COSY spectra, sine-bell window functions in both domains usually give the best combination of resolution, line shape, and signal-to noise. However, components with short spin-spin relaxation values (including lipid and oligopeptides) were better visualized by applying Lorentz-Gauss window functions or by shortening the time domain (Delikatny et al. 1991). This was first shown with a 2D COSY dataset of cultured melanoma cells processed under various conditions. Each cell line and tissue type (and the molecules of interest) must be considered independently when acquiring, analyzing, and presenting 2D MR spectra. The methods are integral to post processing data recorded on a clinical MR scanner that is now both diagnostic and prognostic.

It was this advancement that eventually identified the origin and likely assignment of the resonance with the long T2 relaxation value in the spectral region (Lean et al. 1991, 1992). The origin was fucosylated glycans shown by Hakamori (Hakomori 1985) to be aberrant fucosylation, particularly of Lewis antigens like sialyl Lewis X, associated with tumor progression, metastasis, immune suppression, and resistance to chemotherapy (Blanas et al. 2018).

## Evidence the narrow ^1^H MR signals from malignant cells are not from intracellular lipid droplets

Using a 2D COSY protocol, two malignant Chinese hamster ovary (CHO) cell lines, the EOT and the parental line WT, were studied (Mackinnon et al. 1989). The EOT cells generated a more highly resolved lipid spectrum than the WT cells, a result which correlates with slightly increased levels of triglyceride measured chemically in their highly purified plasma membranes. The intracellular lipid droplets of both lines were measured using fluorescence and electron microscopy. No significant differences were observed. Together, these results provided further evidence that narrow ^1^H MR signals in malignant cells arose from neutral lipid in the plasma membrane, rather than from intracellular lipid droplets.

## Chemical proof of esterified cholesterol and triglyceride in plasma membranes

The chemical composition of highly purified plasma membrane preparations from this series of CHO cell lines, with increasing levels of tumorigenicity was analyzed. Triglycerides (33–41 nmol/mg total lipid) and cholesteryl ester (226–271 nmol/mg) were recorded in the plasma membranes (Mackinnon et al. 1992). The most significant difference was a gradual decrease in the free cholesterol from wild type (312 nmol/mg total plasma membrane lipid), to EOT (204 nmol/mg total plasma membrane lipid), with a concomitant increase in the degree of saturation of the cholesteryl ester fatty acids, particularly palmitic acid. There were no statistically significant differences in the lipid chemical composition of the whole cells in this series.

The one-dimensional ^1^H MR spectra of the four malignant cell lines showed a gradation in intensity of lipid resonances, in the order from wild type to EOT. Notably, the increase in acyl chain signal intensities in the ^1^H MR spectra of this series of CHO cells and emergence of signals from cholesterol and/or cholesteryl ester coincided with alterations in the amount of free cholesterol and the degree of saturation of the fatty-acyl chain of the esterified cholesterol in the plasma membranes. Thus, there was significant evidence that the types of molecules that would associate and tumble isotropically on the MR timescale were neutral lipids.

## The new membrane model is proposed in 1988

A new membrane model was proposed in 1988 (Mountford and Wright 1988) to account for the following:

1. Neutral lipids make up over 6% of the lipid content of plasma membranes from malignant cells.
2. Spectral evidence that cholesteryl ester and triacylglycerol were present in the plasma membranes of precancerous and cancerous cells (Mackinnon et al. 1992).
3. The physics results supported isotropic molecular motion to arise from small structures which retained their integrity within the plasma membrane or associated with it for a sufficiently long time to tumble isotropically. They were distinct from those of the bilayer membrane lipids (Bloom et al. 1986; Mackinnon et al. 1989).
4. The 2D COSY data from the cells bore remarkable similarities and T2 relaxation values to isolated lipoproteins (Williams et al. 1985).
5. That the domains form in the plasma membrane when the cholesteryl ester has a largely saturated fatty acid content (Mackinnon et al. 1992). They also contained ether-linked neutral glycerides (May et al. 1992).

**Fig. 2:**
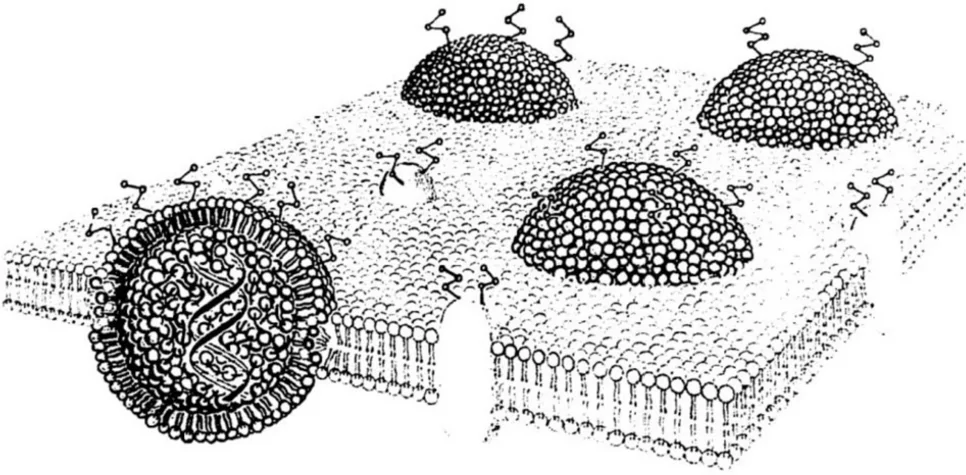
The membrane model proposed in 1998 incorporated neutral lipid domains (Mountford and Wright 1988), where triglyceride, cholesterol, and cholesterol ester, which tumble isotropically and hence mobile on the NMR timescale, are intercalated with phospholipid in the bilayer configuration. The antenna-like appendages attached to the neutral lipid bulges and globular proteins represent cell surface carbohydrate particularly the fucoslyated molecules thought at the time to be the Le^y^ and Le^x^ antigens

The new model (Fig. 2) has neutral lipid domains intercalated with the bilayer lipid of the plasma membrane. A functional role for these neutral lipid domains was also proposed based on plasma membrane alterations which occur with cellular stimulation, with the acquisition of resistance to anti-cancer drugs (May et al. 1992; Mackinnon et al. 1993).

There was no evidence that membrane rafts, which are considered to be dynamic microdomains within cell membranes enriched in cholesterol and sphingolipids, are part of this structure. Rafts play a crucial role in compartmentalizing cellular processes and regulating signaling pathways (Nieto-Garai, Lorizate et al. 2022) .

## Paraskeva model of tumor development

The model developed by Dr. Christos Paraskeva and colleagues is a collection of human colorectal cell lines used to study tumor progression in colorectal cancer. It included both non-tumorigenic adenoma cell lines and tumorigenic carcinoma cell lines. This model allowed the investigation of molecular and cellular changes that occurred during the “transition” from normal cells to cancerous cells. The term “In Transition” has become an important descriptor for documenting “Risk Level.”

The aim was to determine whether MRS (the word “nuclear” was dropped due to patient concern) could distinguish human colorectal cells with increasing malignant potential. A gradual reduction in cellular differentiation and an accumulation of genetic alterations from adenoma to carcinoma characterized the selected cell lines. One-dimensional MRS and 2D COSY showed that reduced cellular differentiation in the model correlated with an increase in the levels of lipid, metabolites, the glycosylation intermediate uridine diphospho-*N*-acetylglucosamine, and cell surface fucosylation (Lean et al. 1992; Mackinnon et al. 1994).

Mutations in K-*ras*, APC, and DCC genes were identified in the adenoma and in the carcinoma cell lines in this model. However, the first evidence of an abnormality in the *p53* gene was concomitant with the cells’ ability to grow as a tumor in athymic nude mice. This genetic change coincided with the detection, by MRS, of UDP-GlcNAc cross peak between H2 at 4.38 ppm and HI at 5.99 ppm, and the appearance of an additional fucosyl resonance (cross peak between -CH_3_ at 1.41 and H5 at 4.30 ppm) in the least tumorigenic cell lines (Mackinnon et al. 1994).

An increase in complexity of the fucosylation spectral pattern was observed with further cellular de-differentiation and increased tumorigenicity (Mackinnon et al. 1994). These data were the first to document the chemical changes in the adenoma-carcinoma sequence measured by MRS. The biomarkers of the tumor development were molecules active on the MR timescale and included lipids, free cholesterol, cholesterol ester, and some metabolites and then choline and fucosylated species (Mackinnon et al. 1994). These are known collectively as “In Transition.”

The observation that the increase in complexity of the fucosylation spectral pattern was reporting on cellular de-differentiation and was diagnostic was confirmed on human biopsies and correlated with the histopathology by Peter Russell (Mackinnon et al. 1995).

## Independent evidence in 2010 of stable, disordered, isotropic, mobile triglyceride aggregates at the center of a planar lipid bilayer

**Fig. 3:**
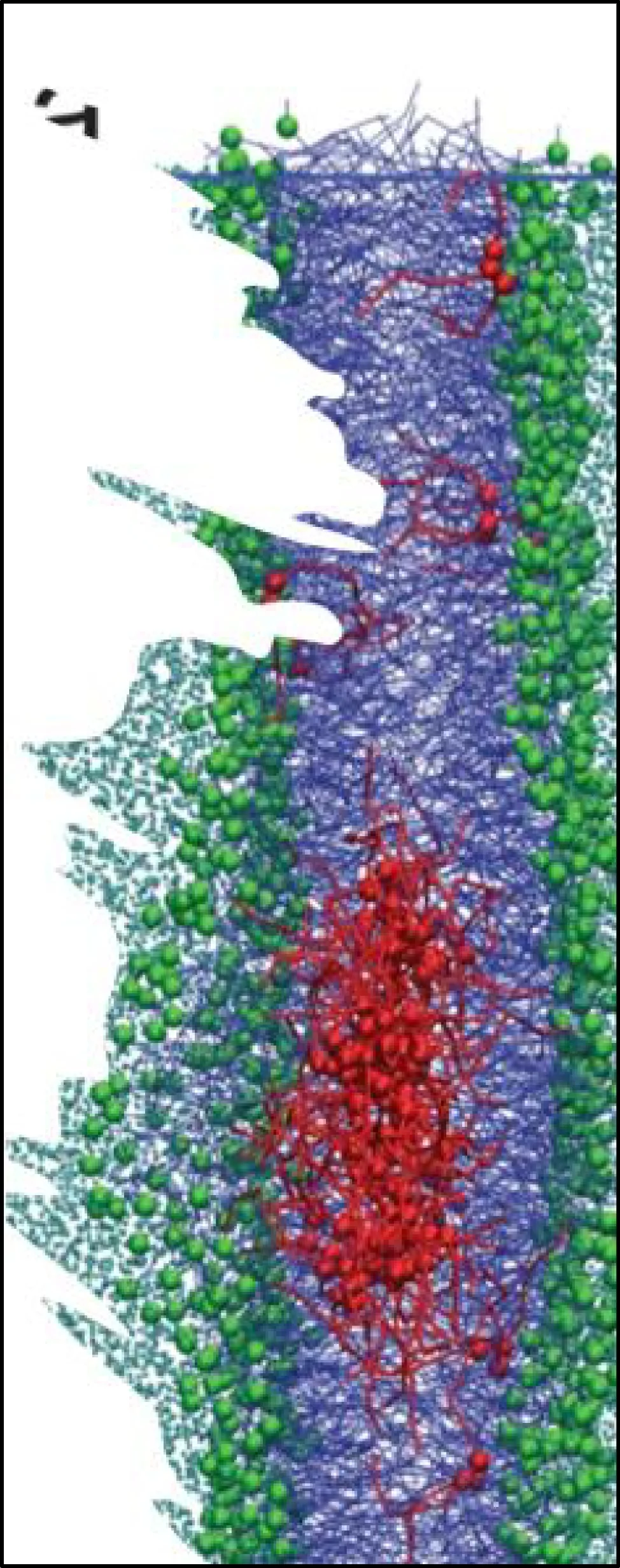
Triglyceride, present above 5%, can be accommodated in a phospholipid bilayer. Abbreviations triolein (TO) also known as triglyceride; palmitoyl-2-oleoyl-*sn*-glycero-3-phosphocholine as the model phospholipid. Phospholipid tails are shown in blue, phospholipid head groups are shown as green spheres, triglyceride or triolein tails are shown in red, triglyceride glycerol backbone is shown as red spheres, and water is depicted in cyan. The blue lines represent the central simulation cell boundaries; with permission (Khandelia et al. 2010)

The membrane model, first proposed in 1988, suggested the presence of isotropically tumbling domains composed of triglycerides, cholesterol, and cholesterol ester. This model was verified in 2010 by a team of Danish scientists, Himanshu Khandelia, Lars Duelund, Kirsi Pakkanen, and John Ipsen (Khandelia et al. 2010). Shown in Fig. 3 are the simulations providing evidence for the formation of stable, disordered, isotropic, mobile triolein aggregates at the center of a planar lipid bilayer, at concentrations of ∼5% and above. In biological systems, similar aggregates, but of a larger size, can be observed in activated or malignant mammalian cells. Their results provide evidence for the existence of the “mobile lipid domain” enriched in neutral lipids in activated cells.

## Clinical importance

New 2D MR pulse sequences have been designed to overcome specific problems associated with the crowded spectra of biological specimens (Ramadan and Mountford 2009) and human organs in vivo (Ramadan and Mountford 2009). This protocol is used today for a range of in vivo applications in a clinical MR scanner (Mountford 2009, Thomas et al. 2009; Ramadan et al. 2011, 2012). The importance of this pulse sequence is demonstrated by its ability to record changes in lipid behavior and composition and to enable correlation of detailed chemical differences recorded in vivo with clinical outcomes.

The lipids, mobile on the MR timescale, were shown to distinguish between the healthy breast tissue of women at no genetic or hereditary risk of breast cancer and that recorded from women with the BRCA gene mutations (Ramadan et al. 2015). While healthy, not at risk breast tissue exhibits lipid-enriched spectra, differences in chemical composition can be detected using the in vivo 2D COSY methods (Thomas et al. 2009; Ramadan et al. 2012) consistent with the observations made in the cell models (Mackinnon et al. 1992, 1994). The same method was reported to be able to independently determine breast density without the need for contrast agent (Santamaría et al. 2022). This is an important finding as it is now recognized that gadolinium contrast agents can build up in the body, including tissues like the brain, bones, and skin, even in people with normal kidney function (Semelka and Ramalho 2023).

In 2025, a 10-year blinded clinical study involving over 400 women at risk for breast cancer was completed. The in vivo 2D COSY method, combined with a series of advanced post processing techniques, accurately predicted that just under 10% who would go on to develop a breast cancer by evaluating their breast tissue chemistry in a clinical 3-T MRI scanner. The tumors were not identified using contrast-enhanced MRI until an average of 4 years later (submitted). The earliest of these detectable chemical changes was lipid that is mobile on the MR timescale and is now documented and known as the first change. All women carrying the BRCA gene mutations have this first lipid change (Ramadan et al. 2015).

## Therapy to prevent transitional changes progressing to a malignant tumor

Statins have the potential to reduce an increase in lipid and cholesterol. All BRCA gene carriers have the first level of transition which is increased levels of triglycerides and cholesterol (Ramadan et al. 2015). Thus, the concept of using statins to prevent this from progressing further in all high-risk women is a working hypothesis. There is recent literature that supports this approach.

Statins historically are used to reduce high cholesterol levels in the blood, specifically low-density lipoprotein cholesterol. They assist in lowering triglycerides and increase high-density lipoprotein cholesterol. Recent reports suggest that statins suppress the mevalonate pathway, reducing tumor cell proliferation, inducing apoptosis, and generating a microenvironment, which assists with targeted therapies (Kamal et al. 2024; Karimi Jirandehi et al. 2024; Tang et al. 2024). How do these isotropically tumbling lipid domains in the plasma membranes of the human respond to statins? This is a new area of research.

## In summary

The early work of Australian and Canadian scientists paved the way for a new diagnostic and prognostic technology to determine the risk for cancer. From the early work, a new membrane model was proposed that included isotropically tumbling neutral lipids intercalated in the phospholipid bilayer of a human cancer cell membrane. Over two decades later, the Danish team confirmed triglycerides in a lipid bilayer above a concentration of 5%.

These neutral lipids, as they appear on the MR timescale, have been shown in vivo on a clinical MRI scanner to constitute the first of a series of transitional changes as cells cease to be normal and are in a series of transitions to a malignant cancer (Mackinnon et al. 1994). The importance of this in management of women at risk for breast cancer has been demonstrated for women carrying the BRCA gene mutations and the results of a 10-year blinded longitudinal study (submitted).

It took 22 years for the membrane model to be verified and some still question it. As Max Planck wisely observed:

“A scientific truth does not triumph by convincing its opponents and making them see the light, but rather because its opponents eventually die and a new generation grows up that is familiar with it.” **Max Planck**

## Supplementary Information

Below is the link to the electronic supplementary material.

ESM 1 (DOCX 13.8 KB)

## Data Availability

No datasets were generated or analysed during the current study.

## Declarations

CM has patents in the subject matter of this presentation and co-founded the scaleup company, DatChem Pty Ltd.
