# Supplementary material for: Controversial membrane structure, independently verified after 22 years, is diagnostic and prognostic for cancer risk
Source: Biophys Rev. 2026 Jun 22;18(4):739–46. doi: 10.1007/s12551-026-01449-4 (PMC13694647; doi:10.1007/s12551-026-01449-4)
Supplement: Supplementary file 1 [file 12551_2026_1449_MOESM1_ESM.docx]

**Vale Ian CP Smith**

One simply cannot describe Ian’s enthusiasm and dedication in one paragraph. We in Australia reported unexpected NMR resonances, from human specimens, that looked to be from a membrane. Ian took these results very seriously whereas most people on the planet did not. He was told I was the “Crazy Lady”. Ian co-opted the best biophysicists to tackle the problem. It was turned into an adventure and is described in the article below. He turned the “Crazy Lady” title into a mark of respect with the physicists gleefully determined to find the solution. At Myer Bloom’s retirement symposium, our multicontinental foray into medical research was clearly a stimulating and important part of all of our careers. The intellectual contributions and friendships that came from the collaboration between Ottawa, Winnipeg, Vancouver and Sydney were all thanks to Ian.

-Carolyn Mountford
